# Structure of the Lassa virus glycan shield provides a model for immunological resistance

**DOI:** 10.1073/pnas.1803990115

**Published:** 2018-06-25

**Authors:** Yasunori Watanabe, Jayna Raghwani, Joel D. Allen, Gemma E. Seabright, Sai Li, Felipe Moser, Juha T. Huiskonen, Thomas Strecker, Thomas A. Bowden, Max Crispin

**Affiliations:** ^a^Oxford Glycobiology Institute, Department of Biochemistry, University of Oxford, OX1 3QU Oxford, United Kingdom;; ^b^Division of Structural Biology, University of Oxford, OX3 7BN Oxford, United Kingdom;; ^c^Centre for Biological Sciences and Institute of Life Sciences, University of Southampton, SO17 1BJ Southampton, United Kingdom;; ^d^Big Data Institute, Li Ka Shing Centre for Health Information and Discovery, Nuffield Department of Medicine, University of Oxford, OX3 7LF Oxford, United Kingdom;; ^e^Helsinki Institute of Life Science and Molecular and Integrative Biosciences Research Program, University of Helsinki, 00014 Helsinki, Finland;; ^f^Institute of Virology, Philipps Universität Marburg, 35043 Marburg, Germany

**Keywords:** Lassa virus, arenavirus, glycoprotein, structure, glycosylation

## Abstract

Lassa virus is an Old World arenavirus endemic to West Africa that causes severe hemorrhagic fever. Vaccine development has focused on the envelope glycoprotein complex (GPC) that extends from the virion envelope. The often inadequate antibody immune response elicited by both vaccine and natural infection has been, in part, attributed to the abundance of *N*-linked glycosylation on the GPC. Here, using a virus-like−particle system that presents Lassa virus GPC in a native-like context, we determine the composite population of each of the *N*-linked glycosylation sites presented on the trimeric GPC spike. Our analysis reveals the presence of underprocessed oligomannose-type glycans, which form punctuated clusters that obscure the proteinous surface of both the GP1 attachment and GP2 fusion glycoprotein subunits of the Lassa virus GPC. These oligomannose clusters are seemingly derived as a result of sterically reduced accessibility to glycan processing enzymes, and limited amino acid diversification around these sites supports their role protecting against the humoral immune response. Combined, our data provide a structure-based blueprint for understanding how glycans render the glycoprotein spikes of Lassa virus and other Old World arenaviruses immunologically resistant targets.

## Significance

Lassa virus is a highly pathogenic arenavirus that causes severe hemorrhagic fever in humans. Currently, there are no efficacious vaccines or treatments available to combat this pathogen. An important component of any vaccine candidate against Lassa virus will likely include the highly glycosylated glycoprotein complex presented on the virion surface. Here, we determine the composition of the Lassa virus glycome, revealing that the virus presents an abundance of glycans that are not biosynthetically processed to full maturity. Such underprocessed glycans form spatially distinct clusters, which shield the proteinous surface of the Lassa virus glycoprotein spike from the humoral immune response. These data are integral for the development of humoral-based vaccines that mimic the mature Lassa virion.

Lassa virus (LASV), an arenavirus endemic to rodent populations in West Africa, causes severe hemorrhagic fever upon transmission to humans, with an estimated 300,000 infections and several thousand deaths each year (1). To date, no licensed vaccine exists against LASV and there are no approved therapeutics for treatment, although the compassionate use of the antiviral drug ribavirin alone, or in combination with favipiravir, has shown efficacy if administered early in infection (2). The inconsistent induction of potent LASV-specific antibody immunity, both during natural infection and by vaccination attempts (3–7), is thought to be partly due to the extensive glycosylation presented on the surface of the LASV glycoprotein complex (GPC) (8). While the architecture of the protein components of LASV GPC is well understood (9–12), structural knowledge of the immunologically important glycans displayed on the GPC surface has remained elusive.

The arenaviral GPC is encoded by the S segment of the single-stranded, bisegmented RNA genome. Following expression of a single polyprotein precursor, the GPC is proteolytically processed by the host cell subtilase SKI-1/S1P (13) and displayed on the lipid bilayer surface of the mature virion as a trimer of heterotrimers (11, 14). Each heterotrimer consists of a receptor-binding GP1 domain (10, 15–19), a GP2 class I membrane fusion protein (10, 19–21), and a retained myristoylated stable signal peptide (SSP), which is required for GPC processing and function (22–24). Host cell entry of Old World arenaviruses and Clade C New World arenaviruses is initiated by GPC-mediated attachment to O-mannose glycans containing xylose-glucuronic acid sugar repeats presented on the cell surface receptor, α-dystroglycan (α-DG) (25–28). Factors including the lowered pH associated with entry into the endocytic pathway, lysosome-associated membrane protein 1 (LAMP1) recognition, and dissociation of GP1 from the GPC are responsible for triggering fusion of the viral envelope and endosomal host cell membrane (11, 29, 30).

The 11 potential *N*-linked glycosylation sites (PNGs) found on each SSP−GP1−GP2 protomer accounts for nearly 30% of the total mass of the LASV GPC (10, 31). It has been shown that LASV glycans promote evasion from the neutralizing antibody response and are required for viral fitness (8). Additionally, underprocessed oligomannose-type glycans on LASV GPC augment infection through interaction with the C-type lectin, dendritic cell-specific ICAM-3−grabbing nonintegrin (DC-SIGN) (32). However, the relative abundance and distribution of these underprocessed glycans and fine structure of the LASV glycome have yet to be determined.

Here, using an established virus-like–particle (VLP) system (11, 14), we provide a global and site-specific analysis of the *N*-linked glycosylation presented on LASV GPC. Our analysis reveals extensive global heterogeneity, ranging from highly processed complex-type glycosylation to underprocessed oligomannose-type glycans. Mapping of these glycans onto the structure of the LASV GPC ectodomain reveals that some of these carbohydrates form a dense shield consisting of clusters of oligomannose-type glycans that are likely to be utilized during DC-SIGN−mediated entry. Sequence analysis of LASV isolates reveals a higher incidence of amino acid variation on the surface of the GPC in regions less occluded by *N*-linked glycosylation. Together, these data reveal how host cell-derived glycosylation renders LASV an immunologically challenging target.

## Results

### Glycan Processing of Trimeric LASV GPC.

**Fig. 1.**
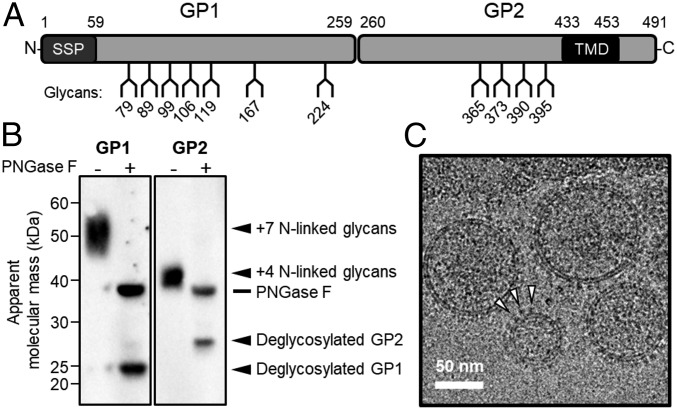
Preliminary characterization of LASV VLPs. (*A*) Schematic representation of LASV GPC, showing the position of *N*-linked glycosylation amino acid sequons (NXT/SX, where X ≠ P), SSP, GP1 attachment glycoprotein, GP2 fusion glycoprotein, and transmembrane domain (TMD). (*B*) Western blot analysis of purified LASV VLPs performed using anti-GP1 (14) and anti-GP2 (34) antibodies, against native and deglycosylated LASV GPC. Blots show LASV GP1 and GP2 before and after PNGase F treatment. (*C*) Electron cryomicroscopy image of VLPs purified from MDCK-II cells. White arrows indicate the positions of prominent LASV GPC spikes. (Scale bar, 50 nm.)

LASV encodes a large number of *N*-linked glycosylation sequons on both the GP1 and GP2 subcomponents of the GPC (Fig. 1*A*). We sought to characterize the composition of the carbohydrate structures displayed on the mature LASV GPC spike using a previously established VLP production and purification strategy, which displays LASV GPC in the full native assembly (11, 14, 33). Western blot analysis of secreted VLPs using an anti-LASV GP1 (14) monoclonal antibody and anti-LASV GP2 polyclonal antibody (34) confirmed the presence and maturation of the GPC into GP1 and GP2 subunits (Fig. 1*B*). Electron cyromicroscopy further verified the expected display of globular GPC spikes on the surface of the purified VLPs (Fig. 1*C*).

**Fig. 2.**
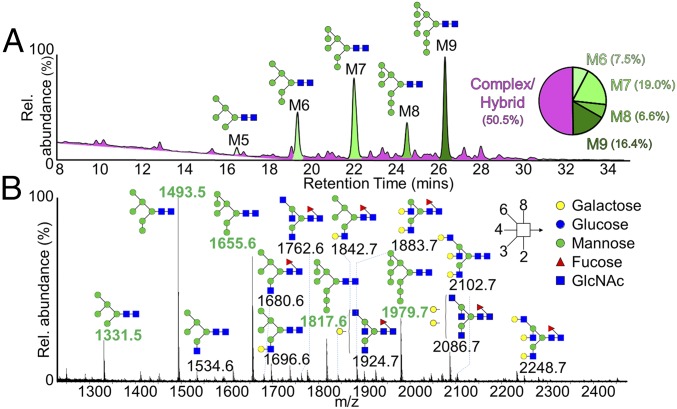
Compositional analysis of LASV GPC glycosylation. (*A*) HILIC-UPLC profile of fluorescently labeled *N*-linked glycans from LASV GPC produced in VLPs. Oligomannose-type glycans (M5 to M9; Man_5_GlcNAc_2_-Man_9_GlcNAc_2_) (green) and complex-type glycans (pink) were identified by Endo H digestion, with quantification of major glycan types summarized as a pie chart. (*B*) Negative-ion electrospray spectrum of *N*-linked glycans from the GPC. Peaks are annotated with the corresponding compositions, using Consortium for Functional Glycomics symbolic nomenclature and Oxford system linkages (65), as per the key. Oligomannose-type glycan *m*/*z* values are labeled in green.

To quantitatively assess the composition of the LASV GPC glycosylation, enzymatically released *N*-linked glycans were fluorescently labeled, and subjected to hydrophilic interaction chromatography−ultra-performance liquid chromatography (HILIC-UPLC) analysis. Cleavage of the released glycan pool with endoglycosidase H (Endo H) revealed an abundance (49%) of oligomannose-type glycans (Fig. 2*A*), particularly Man_6–9_GlcNAc_2_ glycoforms. We observed only a minor population of Man_5_GlcNAc_2_, suggesting the limited processing by Golgi-resident α-mannosidases to convert Man_6_GlcNAc_2_ to Man_5_GlcNAc_2_ at specific sites. The high proportion of oligomannose-type glycans on the LASV GPC is consistent with previous lectin-binding and glycosidase digestion studies (35, 36), which revealed underprocessed glycans on recombinant LASV GP1 constructs, albeit to varying levels.

We ascertained the precise structure of each of the glycans within the released pools by negative-ion mobility−electrospray ionization mass spectrometry (IM-ESI MS) (Fig. 2*B*). Consistent with the UPLC data, IM-ESI MS confirmed a dominant oligomannose-type glycan population, lacking the level of sialylation characteristic to most host cell surface glycoproteins (37, 38).

To confirm the composition of these oligomannose-type glycans, fragmentation by collision-induced dissociation was performed. This analysis reveals the expected array of underprocessed Man_5–9_GlcNAc_2_ species that form following processing by endoplasmic reticulum (ER)-resident α-glucosidases I and II (*SI Appendix*, Fig. S1) (39). Consistent with the biological preference of ER α-mannosidase I to remove the three-branched mannose of the six-antenna (D2) from Man_9_GlcNAc_2_ during *N*-linked glycan processing (40), MS/MS of Man_8_GlcNAc_2_ exhibits a single set of ions derived from a particular (D1, D3) isomer (*SI Appendix*, Fig. S1*B*). We observed two Man_7_GlcNAc_2_ isomers, with the seventh mannose located on either the six-antenna (D3) or three-antenna (D1) arm (*SI Appendix*, Fig. S1*C*). These isomers have also been observed in HIV-1 (gp120) and Semliki Forest virus E1 and E2 glycoproteins (41, 42); it is likely that the existence of high levels of oligomannose glycans among such pathobiologically diverse viruses arises from a requirement for conserved immune evasion and receptor recognition functionality, respectively.

### Structural Clustering of Underprocessed Glycans on the GPC.

**Fig. 3.**
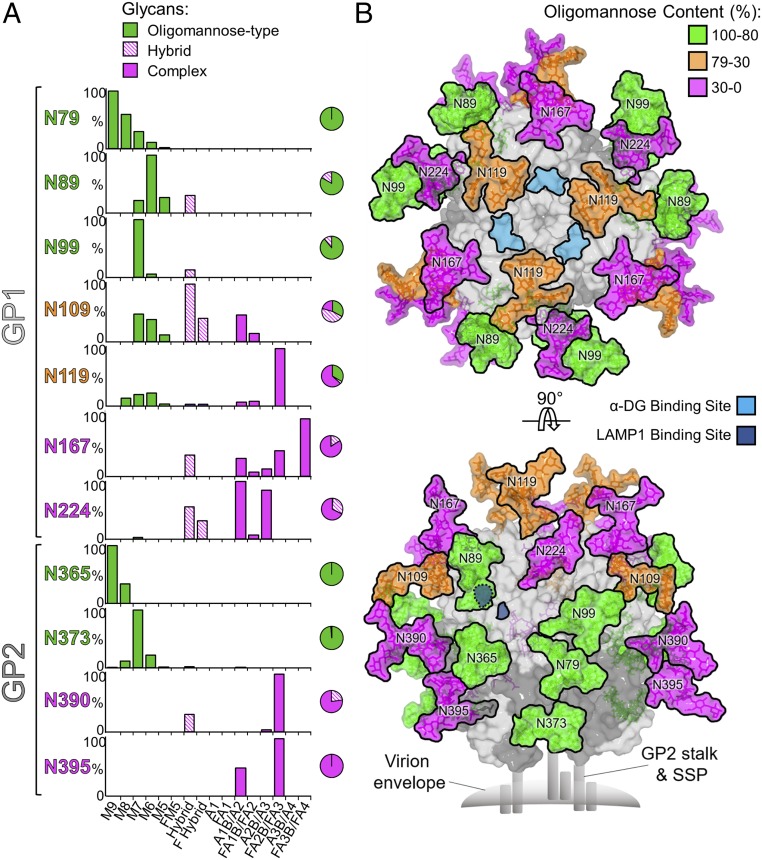
Compositional analysis and structure-based mapping of LASV GPC *N*-linked glycans. (*A*) Quantitative site-specific *N*-linked glycan analysis of LASV GPC. Purified LASV GPC was digested with trypsin, chymotrypsin, and GluC, then analyzed by LC-ESI MS. Glycan compositions are based on the glycan library generated from negative-ion mass spectrometry of released *N*-glycans. The bar graphs represent the relative quantities of each glycan group with oligomannose-type glycan series (M9 to M5; Man_9_GlcNAc_2_ to Man_5_GlcNAc_2_) (green), afucosylated and fucosylated hybrid glycans (Hybrid & F Hybrid) (dashed pink), and complex glycans grouped according to fucosylation and number of antennae (A1 to FA4) (pink). The pie charts summarize the quantification of these glycans. (*B*) Modeling experimentally observed glycosylation onto the crystal structure of the trimeric LASV GPC (PDB ID code 5VK2) (9). The glycans are colored according to the oligomannose content, as defined by the upper-right-hand key. The locations of α-DG and LAMP1 binding sites have been previously proposed (11, 15, 27, 66) and are annotated in cyan and dark blue, respectively. GP1 and GP2 are colored light gray and dark gray, respectively.

It has been previously shown that oligomannose-type glycans displayed on the LASV GPC mediate host cell attachment to DC-SIGN presented on monocyte-derived dendritic cells (32). We sought to identify the possible location(s) of this interaction using an integrated site-specific compositional and structural approach. LASV GPC glycopeptides were generated by digestion with trypsin, chymotrypsin, and GluC and subjected to in-line liquid chromatography−mass spectrometry. This analysis revealed that each site presents a differential level of oligomannose, hybrid, and complex-type glycan populations (Fig. 3*A*). Consistent with our UPLC analysis (Fig. 2*A*), we observed that a number of these sites (Asn79_GP1_, Asn89_GP1_, Asn99_GP1_, Asn365_GP2_, and Asn373_GP2_) were predominantly of the oligomannose type. The level of mannosylation varies widely across these sites, where the glycans at Asn79_GP1_ and Asn365_GP2_ present a dominant population of Man_9_GlcNAc_2_ and Asn89_GP1_, Asn99_GP1_, and Asn373_GP2_ exhibit less restrictive processing and display Man_6_GlcNAc_2_ and Man_7_GlcNAc_2_.

Using the crystal structure of the trimeric LASV GP1−GP2 ectodomain [Protein Data Bank (PDB) ID code 5VK2 (10)], we determined the distribution of the observed glycan populations in a structural context. We generated a model of the fully glycosylated LASV GP1−GP2 ectodomain by overlaying our experimentally determined glycan structures onto crystallographically observed *N*-linked glycosylation sites (Fig. 3*B*). This analysis reveals how *N*-linked glycosylation envelopes the majority of the GPC ectodomain and provides a structure-based model for understanding glycan-mediated shielding of the proteinous LASV GPC surface. At a more detailed level, this integrated structural approach also indicates the existence of a high mannose cluster, which spans GP1 and GP2 subunits. This cluster is located on the side of the GPC trimer and includes Asn79_GP1_, Asn89_GP1_, Asn99_GP1_, Asn365_GP2_, and Asn373_GP2_. We suggest that the dominant oligomannose (94%) composition observed in this region is likely due to the high spatial density of *N*-linked glycans, which limit the accessibility of glycan processing enzymes to these sites. The existence of such a high mannose cluster has precedence and is reminiscent of the “mannose patches” observed on the glycan shield of the HIV-1 envelope glycoprotein (43–45). Further examination reveals that the proposed LAMP1 binding site is partially occluded by this oligomannose cluster. In particular, the glycan extending from Asn89 shields the histidine triad (His92, His93, and His230) of LASV GP1 thought to be a region critical for this interaction (28, 29). The occlusion of this binding site is consistent with the proposed mechanism of LASV GPC−LAMP1 recognition (11, 15, 30), which requires pH-dependent conformational rearrangements of the GPC spike upon exposure to the late endosomes. Indeed, modeling indicates that the formation of the low-pH LASV GP1 conformation redirects the glycan extending from N89 to create a LAMP1-accessible recognition site (*SI Appendix*, Fig. S2).

Restricted glycan processing also occurs at Asn119, albeit to a lesser extent (33% oligomannose composition), at the membrane-distal apex of the trimer (Fig. 3*B*). Interestingly, Asn119 is located near, but does not occlude, residues proposed to be required for α-DG binding (His141, Asn146, Phe147, and Tyr150) (27), and a lymphocytic choriomeningitis virus GPC N119Q site-directed mutation revealed that the Asn119 glycan protects the α-DG receptor binding site from antibody-mediated neutralization (8). The use of *N*-linked glycosylation to obstruct immunodominant receptor-binding sites has been observed for other highly glycosylated viruses, such as the CD4 binding site on the envelope spike of HIV-1 (46) and the sialic acid binding site on the HA of influenza virus (47).

### Amino Acid Sequence Diversification Occurs at Antibody-Accessible Sites on the GPC.

**Fig. 4.**
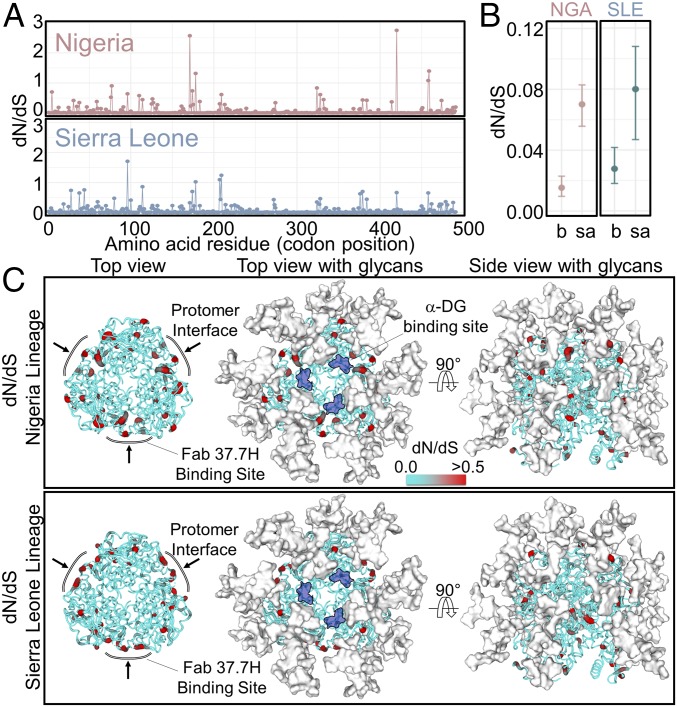
Amino acid sequence diversification across the LASV GPC. (*A*) Site-wise dN/dS analysis of LASV GPC gene sequences from the Nigeria (red, lineage II) and Sierra Leone (blue, lineage IV) lineages. (*B*) Comparison of dN/dS values between buried (b) and solvent-accessible (sa) residues on the LASV GPC for Nigeria (NGA) and Sierra Leone (SLE). The error bars correspond to the 95% highest posterior density intervals, while circles indicate mean dN/dS values. (*C*) Mapping of per residue dN/dS values presented in *A* onto the crystal structure of LASV GPC (PDB ID code 5VK2) (9). LASV GPC is presented as a cartoon with residues colored according to dN/dS values. Residues with elevated dN/dS values are colored in red, and *N*-linked glycans are presented as white surfaces. The putative α-DG binding site is shown in dark blue (27). The interprotomer epitope of Fab 37.7H on LASV GPC is also shown.

The absence of an effective antibody response is a characteristic feature of patients who succumb to LASV infection (3–7). Indeed, extensive affinity maturation is thought to be required to generate tightly binding antibodies capable of targeting and neutralizing the highly glycosylated GPC (48). We sought to quantify the steric role that GPC-displayed glycans play in shielding the GPC surface. Using publicly available LASV GPC gene sequences, we performed a comparative nonsynonymous to synonymous nucleotide substitutions (i.e., dN/dS ratio) analysis of the two major lineages, predominantly found in Nigeria and Sierra Leone (Fig. 4*A* and *SI Appendix*, Fig. S3). Consistent with a recent dN/dS analysis on LASV GPC (49), the mean dN/dS ratios for the Nigeria and Sierra Leone clades were estimated at 0.060 and 0.063, respectively. Using the crystal structure of LASV GPC, we found that solvent-accessible residues were characterized by significantly higher dN/dS (mean dN/dS ratios estimated at 0.070 and 0.080 for the Nigeria and Sierra Leone lineages, respectively), compared with buried residues (mean dN/dS ratios estimated at 0.015 and 0.028 for the Nigeria and Sierra Leone lineages, respectively) (Fig. 4*B*). Interestingly, when mapped onto the structure, amino acids corresponding to nucleotides with the greatest relative number of nonsynonymous substitutions colocalized to regions of the GPC surface with lower levels of glycan density (Fig. 4*C*), distal from the functionally required α-DG binding site (50). These data provide a structural rationale for how protein surfaces occluded by glycan density are shielded from the humoral antibody response of the host and also rationalize previous suggestions that immune selection is a predominant driver of nonsynonymous substitutions for accessible regions of the LASV GPC (49).

**Fig. 5.**
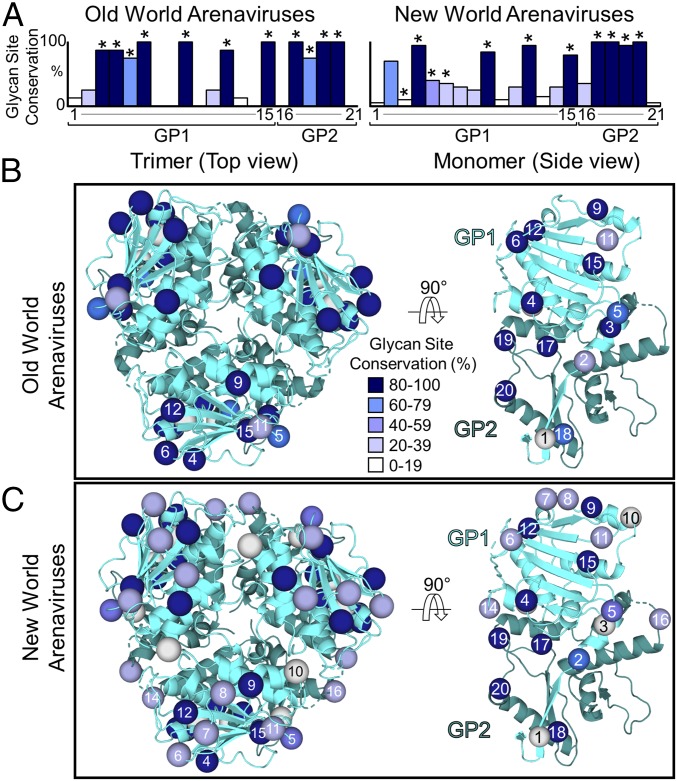
*N*-linked glycosylation site conservation across Old and New World arenaviruses. (*A*) Glycan site conservation across arenaviruses following sequence alignment with Jalview (67). GenBank accession codes for the arenavirus glycoprotein sequences used in this analysis are listed in *SI Appendix*, Fig. S4. Glycan sites are numbered according to the nomenclature established by Sommerstein et al. (8), with those present on LASV GPC labeled with asterisks. (*B* and *C*) The positions of (*B*) Old World and (*C*) New World arenavirus *N*-linked glycosylation sequons mapped onto the structure of LASV GPC (PDB ID code 5VK2) (9). Spheres are colored according to the level of conservation of each glycosylation site.

On a broader level, we note the stringent conservation of LASV GPC *N*-linked glycosylation sequons across Old World arenaviruses, including the five glycan sites that constitute the LASV mannose cluster (Fig. 5*A*), suggesting that mannose patches may be inherent features of Old World arenaviral GPCs. In contrast, the glycan sites constituting the GP1 component of the mannose patch are less prevalent in New World arenaviral GPCs. This juxtaposition may be due to contrasting functional restraints (for example, the requirement to use transferrin receptor rather than α-DG). We suggest that such differential levels of *N*-linked glycosylation site conservation on the GP1 may at least partially account for the contrasting locations of immunodominant neutralizing epitopes on New World (the GP1 subunit) and Old World (GP1−GP2 interfaces) GPCs (10, 18, 51, 52).

## Discussion

While the LASV GPC constitutes a key therapeutic target, vaccine design efforts have been hindered by the host-derived glycosylation that encompasses the protein surface (8). Here, using an established VLP expression strategy, we performed a comprehensive compositional investigation of the LASV glycome. Our LASV GPC was produced in MDCK-II cells and forms a native-like conformation on the surface of the VLP (Fig. 1 *B* and *C*) (11, 14, 33). Although MDCK-II cells do not represent a natural host cell type, due to the stringent conservation of the *N*-linked glycan biosynthesis processing pathway (39) across mammalian cell types (53, 54), we expect only limited variations in the composition and abundance of the *N*-linked glycosylation of LASV GPC. Additionally, we note that the HIV-1 glycome is determined by structural constraints and is compositionally well conserved when produced in varied mammalian cell types (55). We suggest that future studies on the glycosylation state of circulating viruses in LASV-infected patients should corroborate our findings.

We present a structure-based model for rationalizing glycan-mediated shielding of LASV GPC from the humoral host response, revealing a cluster of underprocessed glycans that spans both the GP1 and GP2 subunits of the trimeric spike complex (Fig. 3). While the presence of mannose-rich glycosylation is unusual for cellular proteins, it has often been observed on enveloped viruses, such as orthomyxoviruses, phleboviruses, alphaviruses, and flaviviruses (56). The principles rationalizing the extent of glycan processing of viral proteins have yet to be fully elucidated and should be considered in the context of the route of egress from the host cell. In the case of LASV, however, we suggest that the formation of underprocessed moieties is likely enabled by the local structural environment and that the proximity of glycans extending from Asn79_GP1_, Asn89_GP1_, Asn99_GP1_, Asn365_GP2_, and Asn373_GP2_ sterically impede accessibility to glycan-processing enzymes (*SI Appendix*, Fig. S5). Oligomannose-type glycans were also observed at sites outside of this cluster, albeit at lower levels. The presence of oligomannose at multiple locations across the GPC is suggestive of a promiscuous model of DC-SIGN usage, where numerous *N*-linked glycans may contribute as attachment factors for LASV entry.

The existence of a large population of oligomannose-type structures on LASV GPC has important consequences in the development and production of recombinant vaccine candidates. On recombinant glycoproteins, oligomannose-type glycans are considered to impart elevated clearance rates and may influence immunogenicity (57, 58). In addition, from immunogen design efforts for HIV, we have learned that the levels of oligomannose-type glycans on recombinant mimics of the envelope spike are highly sensitive to the structural constraints imposed by native-like trimeric oligomeric architecture (44, 55). Given the importance of both glycan processing states and native quaternary architecture, it is likely that the current recombinant expression technologies for LASV GP1 (15, 35, 36) and GPC (10) monomeric ectodomains may not reproduce native-like glycosylation. We envisage that the next generation of full-length LASV GPC ectodomain immunogens will both exhibit trimeric architecture and suppress the exposure of natively inaccessible epitopes through the presentation of more-native-like glycosylation.

This study also reveals that amino acid diversification occurs primarily at glycan-devoid regions on the LASV GPC (Fig. 4), providing important clues to what sites may be most vulnerable to the antibody immune response. By analogy to template-based vaccinology approaches against “evasion-strong” pathogens (59), such as HIV-1, it is possible that these regions are viable targets for the development of immunogens capable of effectively neutralizing LASV.

Continued emergence and high pathogenicity render LASV an important human pathogen and underscore the necessity for reagents capable of preventing and treating infection. Given the prominence of *N*-linked glycosylation on the LASV surface, we suggest that the compositional and structural data presented here will be integral for the creation of a humoral immunity-based vaccine capable of mimicking the mature LASV virion.

## Materials and Methods

### Expression and Purification of LASV VLPs.

MDCK-II cells stably expressing LASV GPC (Josiah strain, GenBank ID code AY628203) were cultured in DMEM supplemented with 10% FCS for 96 h. The medium was replaced with DMEM with 2% FCS for VLP expression, as previously described (14). After 96 h, the cell supernatant was cleared by centrifugation (4,000 × *g* for 30 min). VLPs were pelleted through 10 mL of 20% sucrose cushion by ultracentrifugation (170,000 × *g* for 3 h). The pellets were resuspended in SPG buffer at pH 8 for 12 h at 4 °C.

### Western Blot Analysis.

Proteins from LASV VLPs were analyzed by Western blotting, as previously described (11). Monoclonal mouse antibody AC1 against GPC and GP1 and polyclonal rabbit antibody GP4 against GPC and GP2 were used as primary antibodies.

### Electron Cyromicroscopy of VLPs.

A 3-µL aliquot of purified VLP samples was applied on a plasma-cleaned grid coated with holey carbon (C-flat; Protochips). The grids were floated on a drop of 200-µL PBS buffer to remove sucrose and were blotted for 3 s followed by plunge-freezing into a mixture of liquid ethane (37%) and propane (63%) using a plunger device (CP3; Gatan). Micrographs were collected at liquid nitrogen temperature using a 300-kV transmission electron microscope (TF30 Polara; FEI) equipped with a CCD camera (Ultrascan 4000SP; Gatan) at a calibrated magnification of 75,000×, resulting in a final pixel size of 2 Å/pixel.

### Release and Labeling of *N*-Linked Glycans.

Excised LASV GPC gel bands were washed alternately with acetonitrile and water before drying in a vacuum centrifuge. The bands were rehydrated with 100 µL of water and incubated with PNGase F at 37 °C overnight. Released *N*-linked glycans were fluorescently labeled with procainamide. A 100-µL aliquot of labeling mixture (110 mg/mL of procainamide and 60 mg/mL of sodium cyanoborohydrate in 70% DMSO and 30% glacial acetic acid) was added to the sample and incubated at 65 °C for 4 h. Labeled glycans were purified using Spe-ed Amide 2 columns (Applied Separations), as previously described (43).

### Glycan Analysis by HILIC-UPLC.

Labeled glycans were analyzed using a 2.1 mm × 10 mm Acquity BEH Glycan column (Waters) on a Waters Acquity H-Class UPLC instrument as performed in ref. 43, with wavelengths *λ*_ex_ = 310 nm and *λ*_em_ = 370 nm. Endo H digestions of labeled glycans were used to measure abundance of oligomannose-type glycans, as previously described (43).

### Mass Spectrometry of Glycans.

IM-ESI MS and tandem MS of released *N*-linked glycans were performed on a Synapt G2Si instrument (Waters) as previously described (44). Glycans were purified on a Nafion membrane before injection. Data acquisition and processing were carried out using MassLynx v4.11 and Driftscope version 2.8 software (Waters).

### Mass Spectrometry of Glycopeptides.

Aliquots of 150 μg to 200 μg of the VLPs were proteolytically digested using trypsin, chymotrypsin, and GluC (Promega). ProteoExtract Glycopeptide Enrichment Kit (Merck Millipore) was used to enrich glycopeptides for LC-ESI MS. For site-specific analysis of LASV GPC glycosylation, enriched glycopeptides were analyzed on an Orbitrap Fusion Tribrid mass spectrometer (Thermo Fisher Scientific) coupled to an EASY-Spray nano-LC system (Thermo Fisher Scientific). MS data were acquired with XCalibur 4.0 (Thermo Fisher Scientific). Glycopeptides were fragmented using both higher-energy collisional dissociation and energy transfer dissociation. Data analysis and glycopeptide identification were performed using Byonic and Byologic software (Protein Metrics).

### Phylogenetic and Molecular Evolution Analysis.

Publicly available sequences encoding the GPC spike were downloaded from GenBank and manually aligned. The sequence alignment comprised 217 sequences, spanning 1,473 bp of GPC region. A maximum likelihood phylogeny was inferred using randomized axelerated maximum likelihood (RAxML), where the model parameters included general time reversible model with rate categories (GTRCAT) substitutional model and rapid bootstrap analysis with 100 bootstrap samples (60). The resulting phylogeny indicated two distinct lineages that were supported by high bootstrap score (i.e., >0.95). Specifically, one lineage was dominated by sequences from Nigeria, while the other was predominantly observed in Sierra Leone. Since these two lineages likely represent independent epidemics, we estimated site-wise dN/dS ratios and mean amino acid diversity for each lineage. For the dN/dS analysis, Bayesian molecular clock phylogenies were estimated using Bayesian evolutionary analysis and sampling trees (BEAST) v1.8.4 (61). We assumed a strict molecular clock, Bayesian Skyline coalescent prior, and a codon-structured substitution model (62). Multiple independent Markov chain Monte Carlo (MCMC) runs of 10 million steps were executed to ensure stationarity and convergence had been achieved. An empirical distribution of 4,500 molecular clock phylogenies was obtained by combining (after the removal of burn-in) the posterior tree distributions from the separate runs, used to estimate dN/dS ratios using renaissance counting (63) implemented in BEAST v 1.8.4. Site-wise mean amino acid diversity was calculated using the Python script shown in *SI Appendix, Supplementary Method*.

### Model Construction.

Structural models of *N*-linked glycan presentation on LASV GPC were created using the trimeric crystal structure [PDB ID code 5VK2 (10)], complex-, hybrid-, and high mannose-type *N*-linked glycans (PDB ID codes 4BYH, 4B7I, and 2WAH, respectively). The most-dominant glycoform presented at each site, as observed on LASV GPC when produced in MDCK cells, was modeled onto the *N*-linked carbohydrate attachment sites in Coot (64).

## Supplementary Material

Supplementary File
