# Supplementary material for: Structure of the Lassa virus glycan shield provides a model for immunological resistance
Source: Proc Natl Acad Sci U S A. 2018 Jun 25;115(28):7320–5. doi: 10.1073/pnas.1803990115 (PMC6048489; doi:10.1073/pnas.1803990115)
Supplement: Supplementary File [file pnas.1803990115.sapp.pdf]

Supplementary Figures and Methods for:

Structure of the Lassa Virus Glycan Shield Provides a Model for Immunological Resistance

This document includes Supplementary Figures S1-S5, Supplementary Figure Legends, Supplementary Methods and Supplementary References.

www.pnas.org/cgi/doi/10.1073/pnas.1803990115

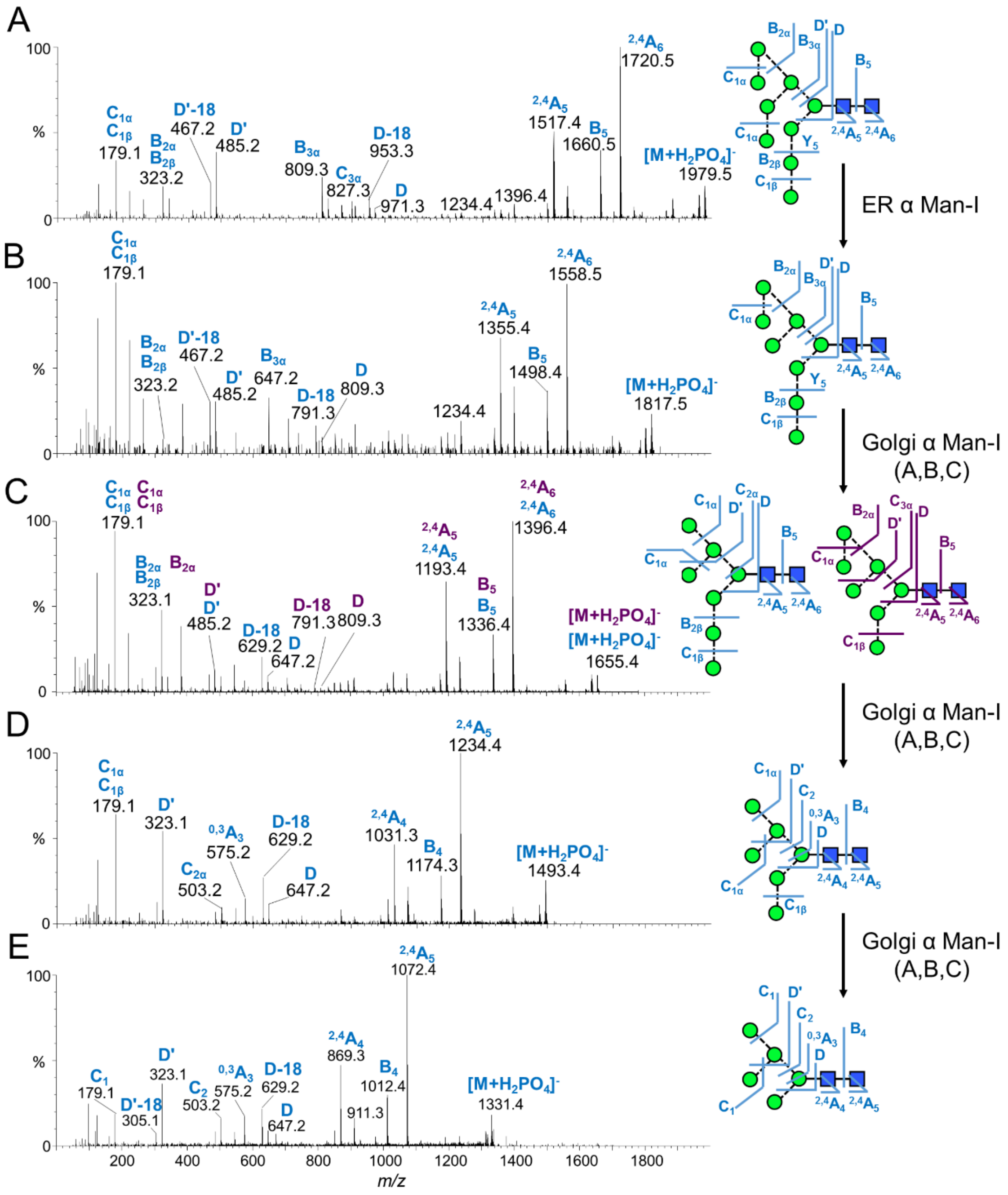

Fig. S1. Mobility-extracted negative-ion collision induced dissociation tandem MS spectra of oligomannose-type glycans from LASV GPC. (A) $Man_9GlcNAc_2$ (B) $Man_8GlcNAc_2$ (C) $Man_7GlcNAc_2$ (D) $Man_6GlcNAc_2$ (E) $Man_5GlcNAc_2$. Arrows indicate the direction of biological glycan processing.

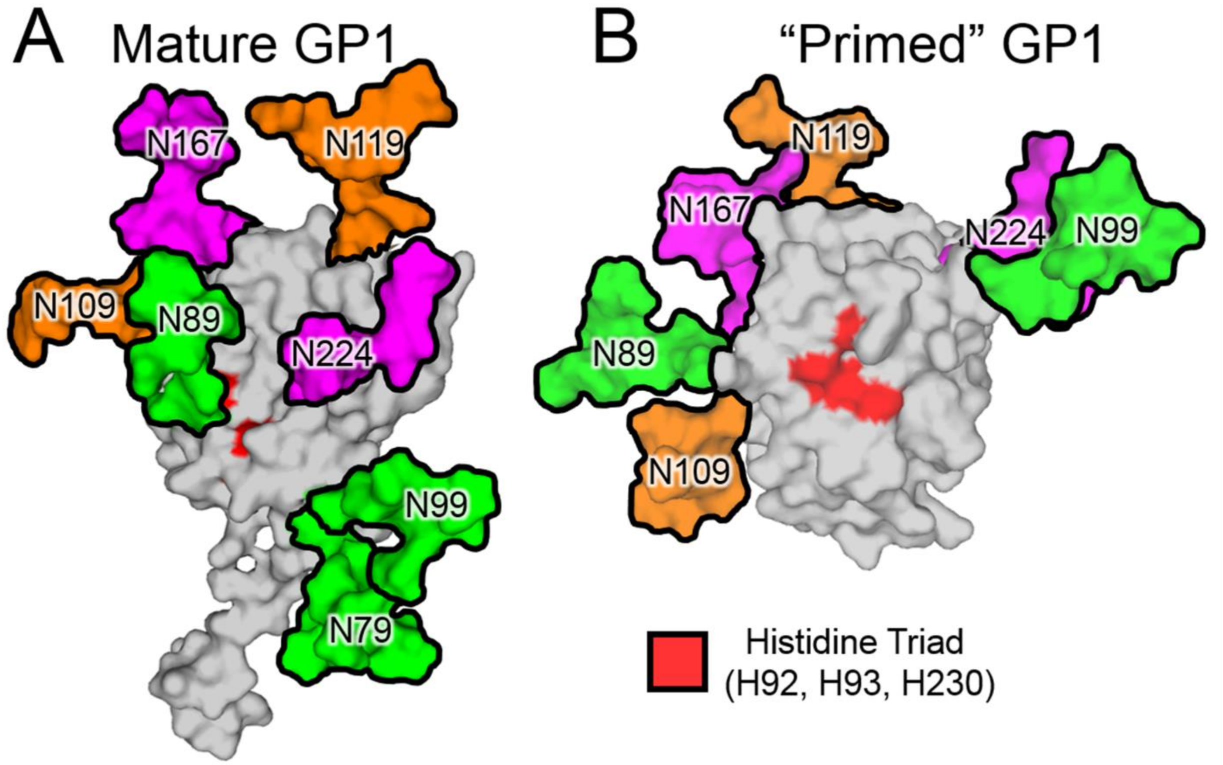

Fig. S2. The glycan extending from N89 partially obstructs the putative histidine triad on LASV GP1 in the prefusion state and is made accessible upon 'priming' (1) of the glycoprotein. (A) Occlusion of the LAMP-1-binding histidines (red) by N89 in the LASV prefusion state (PDB ID: 5VK2 (2)). (B) N-linked glycans mapped onto low-pH conformation of LASV GP1 (PDB ID: 4ZJF(1)).

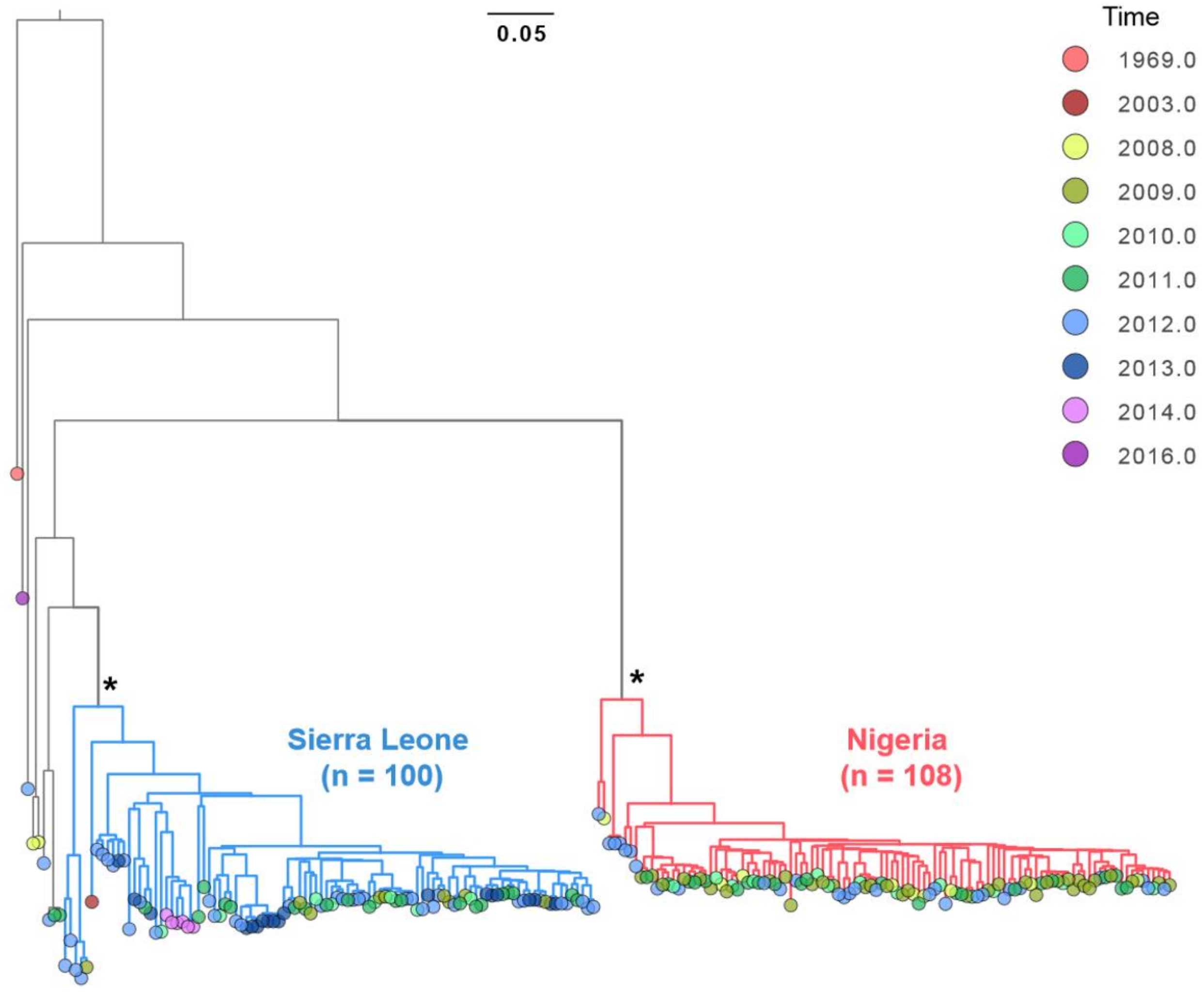

Fig. S3. Maximum likelihood phylogeny of Lassa virus using GPC gene sequences (n=217). Two main lineages are observed, one predominantly found in Nigeria (Lineage II, red), while the other is mainly found in Sierra Leone (Lineage IV, blue).

	Arenavirus	Clade	Acronym	Genbank	No. glycans	GP1															GP2					
						1	2	3	4	5	6	7	8	9	10	11	12	13	14	15	16	17	18	19	20	21
	Lassa	-	LASV	J04324	11			79	89	99	109			119			167			224		365	373	390	395	
	Ippy	-	IPPXV	DQ328877	14	66		78	88	97	107			117		159	165	197		228		370	378	395	400	
Old World	Mobala	-	MOBV	AY342390	11			78	88	98	108			118			166			224		366	374	391	396	
	Mopeia	-	MOPV	DQ328874	11			78	88	98	108			118			166			222		364	372	389	394	
	Morogoro	-	MORV	EU914103	11			78	88	98	108			118			166			222		364	372	389	394	
	Dandenong	-	DANV	EU136038	10		80	85	95		114			124			171			232		372		397	402	
	Lymphocytic coriomeningitis	-	LCMV	AY847350	9			85	95		114			124			171			232		372		397	402	
	Lujo	-	LUJV	FJ952384	10		73			93	104			112		148				194		328	336	353	358	
	Pirital	A	PIRV	AF277659	13		75		90	101	112	117	122	133			182	218				381	389	406	411	
	Allpahuayo	A	ALLV	AY012687	12		74		89		111		119	130			179		223	240		379	387	404	409	
	Pichinde	A	PICV	NC006447	15	67	74		89	100	111	116	121	132			181	217		241		380	388	405	410	
	Parana	A	PARV	AF512829	14		74		89		111	116	119	130			179	215	219	240		379	387	404	409	
	Flexal	A	FLEV	AF512831	12		74		89		111	116		130			179		223	240		379	387	404	409	
	Oliveros	C	OLVV	U34248	13		75		90	101	112	117		131			180		234	251		390	398	415	420	
	Latino	C	LATV	AF512830	13		75		90	101	112	117		131	138		180		231	248		387	395		417	
New World	Machupo	B	MACV	NC005078	9			83	95						137	166	178					369	377	394	399	
	Junin	B	JUNV	D10072	8				95		105					166	178					358	366	383	388	
	Tacaribe	B	TCRV	NC004293	8			83	95							164	176					368	376	393	398	
	Chapare	B	CHPV	EU260463	11		69		88	99				125		171	178			218		357	365	382	387	
	Sabia	B	SABV	NC006317	11		69		88	99				125		171	178			222		361	369	386	391	
	Guanarito	B	GTOV	NC005077	10				88					125			174		202	214	314	352	360	377	382	
	Amapari	B	AMAV	AF512834	10				88	99				128			174			214	315	353	361	378	383	
	Cupixi	B	CPXV	AF512832	10				88	99				125			174			214	315	353	361	378	383	
	Skinner Tank	D	SKTV	EU123328	11		73		88					129		168	178			214	316	354	362	379	384	
	Whitewater arroyo	D	WWAV	AF228063	11		73		88					126		165	176			215	315	353	361	378	383	
	Catarina	D	CATV	DQ865245	11		73		88					129			180			217	318	357	365	382	387	415
	Tamiami	D	TAMV	AF512828	11		73		88				117	128			179			218	320	358	366	383	388	
	Bear Canyon	D	BCNV	AF512833	9		73		88					130			179			216		356	364	381	386	

Fig. S4. N-linked glycosylation sites across arenaviral GPCs. Glycan sites were numbered according to the nomenclature established by Sommerstein *et al.* (3), in order after sequence alignments preformed in Jalview (4). For each N-linked glycosylation sequon, the asparagine position is indicated by the residue number in the table.

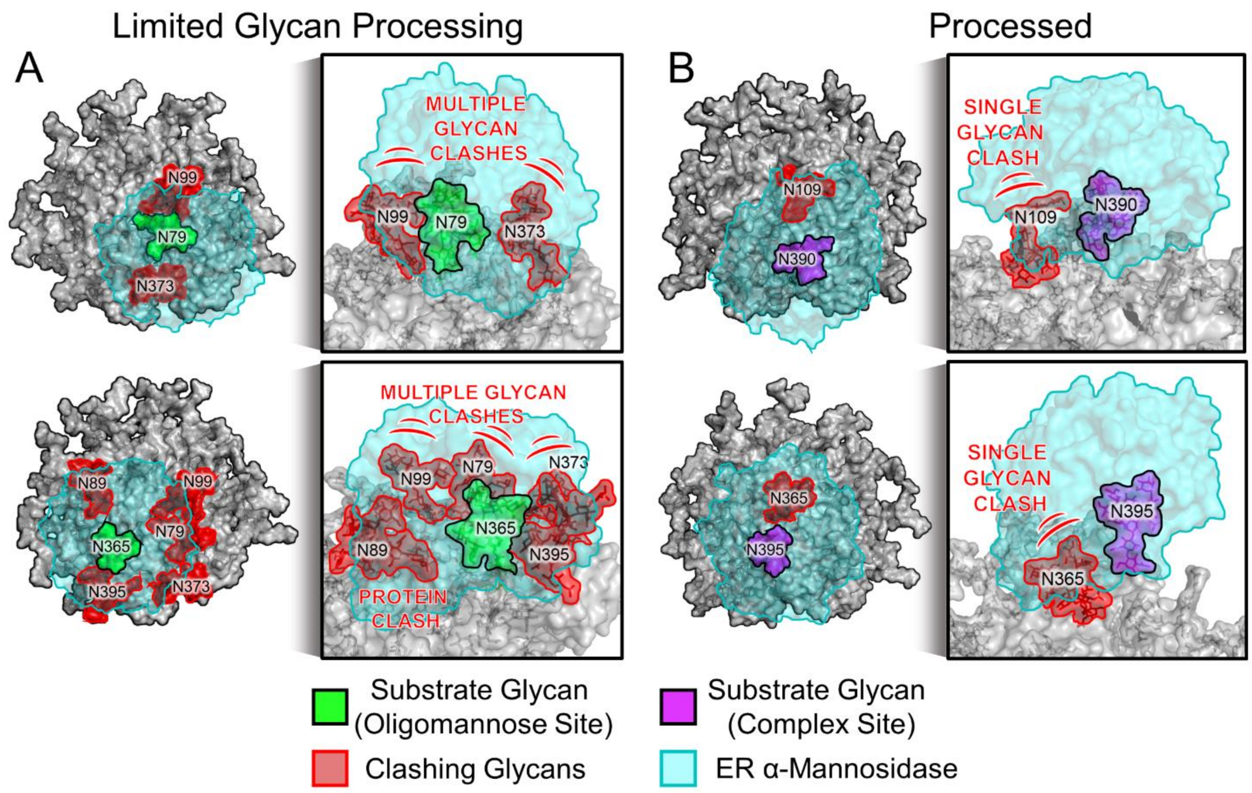

Fig. S5. A model for rationalizing enzymatic resistance to glycan processing on the LASV GPC. (A) The crystal structure (PDB ID: 5KIJ) of ER α1,2-mannosidase I (cyan) was modelled onto the $Man_9GlcNAc_2$ substrate glycans (green) of N79 and N365 of LASV GPC (PDB: 5VK2). Neighboring glycan sites that sterically clash with the enzyme are highlighted in red. (B) Modelling of ER α1,2-mannosidase I (cyan) onto $Man_9GlcNAc_2$ at positions N390 and N395 (pink) revealed only partial clashes with a single neighboring glycan.

Supplementary Method

The following python script was used for site-wise mean amino acid diversity analysis:

```
from Bio import SeqIO
from Bio import AlignIO
import numpy as np
import pandas as pd
import random as r
```

```
from Bio.Seq import Seq
def plot_pairwise_diff(fastain, window_size, sliding_window):
    aln = AlignIO.read('%s'%fastain, 'fasta')
  indices = range(len(aln))
    r.shuffle(indices)
    random_array = []
    for i in range(len(indices)):
        random_array.append(aln[indices[i]])
    random_aln = AlignIO.MultipleSeqAlignment(random_array)
   seq_length = len(random_aln[1,:])
    n_windows = seq_length/window_size

    midpoint = []
    pwd = []
    raw_diff = []
    window_no = []
    count = 1
    end = window_size
    sliding_window_size = 100
    if(sliding_window):
        while end < seq_length:
            #print window_size
            start = (count-1)*sliding_window_size
            print count, start, end, end-start
            if(start >= end):
                break
            midpoint.append(float((start+end)/2))
            sub_aln = aln[:,start:end]
            diff = 0
            x = 0
            for s in range(len(sub_aln)-1):
                seq1 = sub_aln[s,:].seq
                seq2 = sub_aln[(s+1),:].seq
                for b in range(len(seq1)):
                    if(seq1[b] != '-'):
                        if(seq2[b] != '-'):
                            x+=1
                            if(str(seq1[b]).upper()!=str(seq2[b]).upper()):
                                diff+=1
            print "count", x, float(len(sub_aln)), float(x)/float(len(sub_aln)*window_size),
float(diff)/float(x)
            pwd_i = float(diff)/float(x)#float(end-start)/float(len(sub_aln))
            pwd.append(pwd_i)
            raw_diff.append(float(diff) / float(len(sub_aln)))
            end += sliding_window_size
            count += 1
```

```
    else:
     for each in range(n_windows):
            diff = 0
            x = 0
            start = each * window_size
            end = (each + 1) * window_size
            print start, end, each+1
            sub_aln = random_aln[:, start:end]
            midpoint.append(float((start + end + 2) / 2))
            for j in range(len(sub_aln)):
                for k in range(len(sub_aln)-1):
                    seq1 = sub_aln[j, :].seq
                    seq2 = sub_aln[(k + 1), :].seq
                    seq1 = str(seq1).replace('-','N')
                    seq2 = str(seq2).replace('-','N')
                    seq1_aa = Seq(seq1).translate()
                    seq2_aa = Seq(seq2).translate()
                    #print seq1_aa, seq2_aa
                    for b in range(len(seq1_aa)):
                        if (seq1_aa[b] != 'X'):
                            if (seq2_aa[b] != 'X'):
                                x += 1
                                if (seq1_aa[b] != seq2_aa[b]):
                                    diff += 1
                    if x>0:
                print each, float(diff)/x, x
                pwd_i = float(diff)/x
            else:
                pwd_i = float('nan')
            pwd.append(pwd_i)
            raw_diff.append(float(diff)/float(len(sub_aln)))
            window_no.append(each+1)
    print len(pwd), len(raw_diff)
    return {"codon_no":window_no, "mid": midpoint, "pwd":pwd}
#variable called window size, controls how large the windows are
window_size = 3
# sliding window is set to True
sliding_window = False
fastain1 = str("~/lassa_fever_virus/old/clade1_sierraleone.fasta")
fastain2 = str("~/lassa_fever_virus/old/clade2_nigeria.fasta")
nig = plot_pairwise_diff(fastain2, window_size, sliding_window)
nig_d = pd.DataFrame(data=nig)
nig_d.to_csv("~/lassa_fever_virus/aminoacid_diversity_per_site_nigeria_lassafever.csv")
sl = plot_pairwise_diff(fastain1, window_size, sliding_window)
sl_d = pd.DataFrame(data=sl)
sl_d.to_csv("~/lassa_fever_virus/aminoacid_diversity_per_site_sierraleone_lassafever.csv")
```

Supplementary References

1. Cohen-Dvashi H, Cohen N, Israeli H, & Diskin R (2015) Molecular Mechanism for LAMP1 Recognition by Lassa Virus. *Journal of virology* 89(15):7584-7592.
2. Hastie KM, *et al.* (2017) Structural basis for antibody-mediated neutralization of Lassa virus. *Science* 356(6341):923-928.
3. Sommerstein R, *et al.* (2015) Arenavirus Glycan Shield Promotes Neutralizing Antibody Evasion and Protracted Infection. *PLoS Pathog* 11(11):e1005276.
4. Waterhouse AM, Procter JB, Martin DM, Clamp M, & Barton GJ (2009) Jalview Version 2--a multiple sequence alignment editor and analysis workbench. *Bioinformatics* 25(9):1189-1191.
